# Gastrointestinal Cancer Therapeutics via Triggering Unfolded Protein Response and Endoplasmic Reticulum Stress by 2-Arylbenzofuran

**DOI:** 10.3390/ijms25020999

**Published:** 2024-01-13

**Authors:** Kui Zhang, Xin Hu, Jingjing Su, Dong Li, Abhimanyu Thakur, Vikramsingh Gujar, Hongjuan Cui

**Affiliations:** 1 State Key Laboratory of Resource Insects, Medical Research Institute, Southwest University, Chongqing 400715, China; 2 State Key Laboratory of Resource Insects, Institute of Sericulture and Systems Biology, Southwest University, Chongqing 400715, China; 3 Department of Neurosurgery, Massachusetts General Hospital, Harvard Medical School, Boston, MA 02115, USA; 4 Department of Anatomy and Cell Biology, Okhlahoma State University Center for Health Sciences, Tulsa, OK 74107, USA

**Keywords:** *Morus alba* L., anti-cancer bioactive compounds, unfolded protein response (UPR), ER stress, Moracin P, gastrointestinal cancers

## Abstract

Gastrointestinal cancers are a major global health challenge, with high mortality rates. This study investigated the anti-cancer activities of 30 monomers extracted from *Morus alba* L. (mulberry) against gastrointestinal cancers. Toxicological assessments revealed that most of the compounds, particularly immunotoxicity, exhibit some level of toxicity, but it is generally not life-threatening under normal conditions. Among these components, Sanggenol L, Sanggenon C, Kuwanon H, 3′-Geranyl-3-prenyl-5,7,2′,4′-tetrahydroxyflavone, Morusinol, Mulberrin, Moracin P, Kuwanon E, and Kuwanon A demonstrate significant anti-cancer properties against various gastrointestinal cancers, including colon, pancreatic, and gastric cancers. The anti-cancer mechanism of these chemical components was explored in gastric cancer cells, revealing that they inhibit cell cycle and DNA replication-related gene expression, leading to the effective suppression of tumor cell growth. Additionally, they induced unfolded protein response (UPR) and endoplasmic reticulum (ER) stress, potentially resulting in DNA damage, autophagy, and cell death. Moracin P, an active monomer characterized as a 2-arylbenzofuran, was found to induce ER stress and promote apoptosis in gastric cancer cells, confirming its potential to inhibit tumor cell growth in vitro and in vivo. These findings highlight the therapeutic potential of Morus alba L. monomers in gastrointestinal cancers, especially focusing on Moracin P as a potent inducer of ER stress and apoptosis.

## 1. Introduction

Cancer is a leading cause of death worldwide [1], with gastrointestinal cancers such as colon, pancreatic, and gastric cancer posing significant health threats [2]. Despite the extensive efforts directed toward understanding the pathophysiology of these malignancies and developing therapeutic interventions [3], effective treatments are still lacking, and prognosis remains poor for many patients [4]. Gastrointestinal cancers are a diverse group of malignancies that arise from different sites in the digestive system, each with its own unique risk factors, symptoms, and molecular pathology [5]. For instance, colon cancer typically starts as noncancerous polyps that may, over time, turn malignant [6], whereas pancreatic cancer often commences in the cells lining the ducts of the pancreas [7]. Gastric cancer, on the other hand, commonly begins in the lining of the stomach [8]. Despite the differences, these cancers share the feature of being particularly aggressive and hard to treat [2]. Therefore, it is urgent to develop efficient therapeutic agents and strategies for combating gastrointestinal cancers.

Nature has been a prolific source of bioactive compounds with potential anti-cancer properties [9,10]. One such natural source is *Morus alba* L., commonly known as white mulberry, which is a traditional plant that has been used in various pharmacopeias for centuries due to its beneficial health effects [11,12]. It contains a variety of bioactive compounds, including phenolic acids, flavonoids, and anthocyanins [13]. These compounds have been shown to have several potential health benefits, including antioxidant, anti-inflammatory, and anti-cancer effects [14]. In recent years, research into the potential of *Morus alba* L. for treating cancer has been growing. Some studies have shown that the bioactive compounds in white mulberry can inhibit the growth of cancer cells [15,16], induce apoptosis (programmed cell death) [3,17], and even promote cancer cell differentiation [18]. However, the detailed mechanisms of these compounds and their effect on gastrointestinal cancer cells have not been well studied. Thus, more research is required to fully understand how *Morus alba* L. works against cancer and determine its efficacy in clinical trials.

In this study, we, for the first time, evaluated the anti-cancer activity of 30 different monomeric compounds extracted from *Morus alba* L. against gastrointestinal cancer cell lines, including colon, pancreatic, and gastric cancer. It aimed to elucidate the mechanisms through which these compounds exert their anti-cancer effects, focusing on cell proliferation, DNA replication, the unfolded protein response (UPR), and ER stress. Notably, an in-depth analysis of a potent bioactive compound, Moracin P, was conducted to examine its impact on the induction of ER stress and apoptosis in gastrointestinal cancer cells both in vitro and in vivo. By exploring these mechanisms, this research may pave the way for developing novel therapeutic strategies for gastrointestinal cancers, expanding our understanding of how natural compounds can be harnessed in the battle against these malignancies.

## 2. Results

### 2.1. Characterization and Source Distribution of Bioactive Compounds from Morus alba L.

In this study, thirty bioactive monomer compounds, including but not limited to Moracin P, Oxyresveratrol, and Chlorogenic Acid, were extracted from various parts of the *Morus alba* L. tree, namely its roots, branches, bark, leaves, and fruits. Each compound, with its unique chemical structure and functional groups, underscores the rich diversity of bioactive elements within *Morus alba* L., as listed in Table 1. These bioactive compounds primarily encompass five chemical categories: phenolic compounds, stilbenoids, flavonoids, polyphenols, and alkaloids. These phenolic compounds, such as Moracin P and Moracin O, are recognized by their aromatic rings and hydroxyl groups and are primarily found in the tree’s roots and branches. Stilbenoids, which include Oxyresveratrol and Resveratrol, are characterized by two phenyl rings linked by a methylene bridge and are prevalent in the bark and leaves. Flavonoids, like Morin, are marked by a 15-carbon skeleton and are predominantly located in the leaves and fruits. Polyphenols, such as chlorogenic acid, identifiable by their caffeic acid and quinic acid functional groups, are concentrated mainly in the leaves. Meanwhile, alkaloids like 1-Deoxynojirimycin, known for their nitrogen-containing cyclic structures, are found in both the tree’s roots and leaves. Lastly, glycosides, exemplified by Mulberroside A and C, are compounds with a sugar moiety linked to a non-sugar moiety (aglycone) and are typically isolated from the root bark. Table 1 shows a comprehensive summary of these compounds and their distribution within the *Morus alba* L. tree.

### 2.2. Inhibitory Effects of Morus alba L. Compounds on Gastrointestinal Cancer Cells

**Figure 1:**
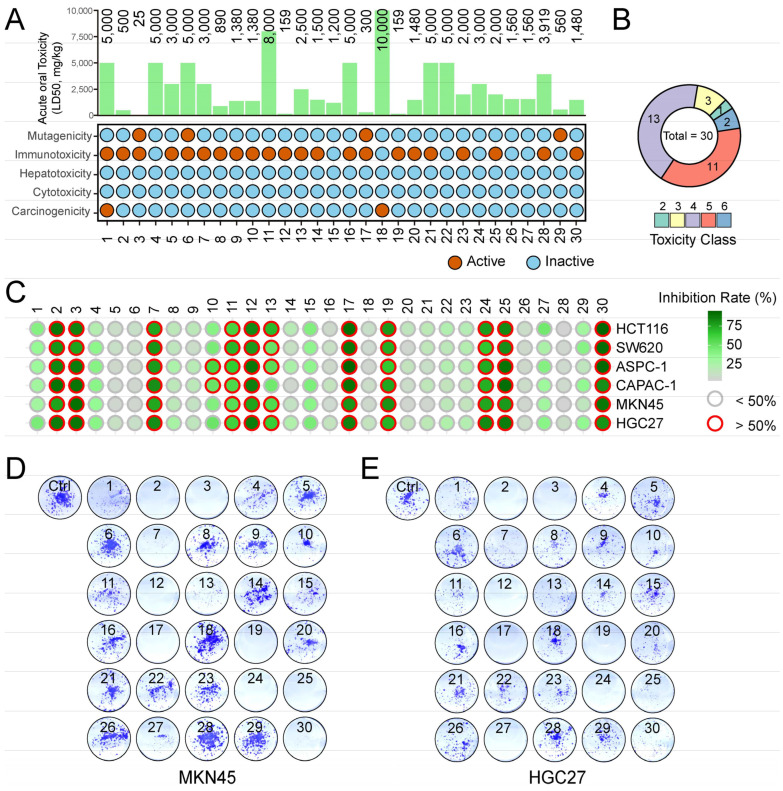
**Screening of bioactive compounds in *Morus alba* L. for anti-cancer activities.** (**A**) The toxicological parameters of 30 bioactive phytochemicals in *Morus alba* L. are analyzed, encompassing acute oral toxicity, hepatotoxicity, and various toxicological endpoints, namely carcinogenicity, immunotoxicity, mutagenicity, and cytotoxicity observed in acute oral exposure. In the represented data, red and blue circles denote active and inactive statuses, respectively. (**B**) The count of phytochemicals within distinct toxicity classes is presented for the active toxicity endpoints among the 30 examined bioactive phytochemicals. (**C**) The dot plot heatmap represents the inhibitory effects of the tested compounds on tumor cell growth. The anti-cancer potential of bioactive compounds extracted from *Morus alba* L. was examined across three different types of cancer: colon (HCT116 and SW620), pancreatic (ASPC-1 and CAPAN-1), and gastric (MKN45 and HGC27). For the treatment, 100 μM of each drug was administered to the respective cancer cells for 3 days, after which the rate of inhibition was calculated. The transition from grey to green color represents a range from low to high inhibition rate, and inhibition rates exceeding 50% are indicated with red circles, while those below 50% are marked in grey. (**D**,**E**) The evaluation of anti-cancer activities was carried out using the plate clone formation assay. MKN45 and HGC27 cells were seeded into plates and exposed to each of the candidate compounds at a concentration of 100 μM for 2 weeks. Cells incubated with an equal volume of DMSO served as the control group.

The analysis of thirty active substances via the ProTox II web server yielded toxicity predictions indicating a predominant manifestation of immunotoxicity among the tested compounds. Notably, eight of these active substances showed no toxicity, each demonstrating no more than two types of toxic effects (Figure 1A). The predicted toxicity classes indicated that the majority of these active substances fell into Classes 4 and 5 (Figure 1B), indicating a relatively low level of toxicity. The presence of the compounds in these classes suggests that while they may exert some level of toxicity, this is not typically life-threatening under normal conditions. In conclusion, the toxicity analysis primarily revealed a predominant occurrence of immunotoxicity and identified eight compounds with negligible toxicity, with these components displaying a maximum of two toxic effects. The majority of the active substances were classified under toxicity Classes 4 and 5, highlighting their lower toxicity levels. These findings provide valuable insights and a solid foundation for further investigation and development of these active substances in various applications, given their relatively low toxicity profiles.

The anti-cancer potential of *Morus alba* L. compounds is evaluated by examining their effects on various types of digestive tract cancer cells, namely colon (HCT116 and SW620), pancreatic (ASPC-1 and CAPAN-1), and gastric (MKN45 and HGC27). Each cell type is exposed to thirty monomer compounds at a concentration of 100 μM, and the growth inhibitory effects are recorded after a treatment period of three days. The results in Figure 1C elucidate the distinct inhibitory effects of the compounds on various cancer cell types. Compounds including Sanggenol L, Sanggenon C, Kuwanon H, Eleutheroside A, 3′-Geranyl-3-prenyl-5,7,2′,4′-tetrahydroxyflavone, Morusin, Morusinol, Mulberrin, Moracin P, Kuwanon E, and Kuwanon A demonstrate robust inhibitory effects on all tested cell types, with inhibition rates exceeding 50%. Furthermore, Oxyresveratrol 3′-O-β-D-glucopyranoside exhibits a selective inhibitory effect, showing an inhibition rate of over 50% on pancreatic cancer cells (ASPC-1 and CAPAN-1) but a lower inhibition efficiency on other cell types. These findings underscore the diverse effects of these compounds and their potential as selective therapeutics for various forms of digestive tract cancers (Figure 1C).

### 2.3. Validation and Further Exploration of Growth Inhibitory Effects Using Plate Clone Assay

The initial screening results were validated and further explored using clonogenic assays, which examined the survival and proliferation of cells following treatment with each compound. These investigations mainly utilized gastric cancer as the representative model. After two weeks of treatment with various compounds, the resulting cell colonies were stained and examined. The findings revealed that Sanggenol L, Sanggenon C, Kuwanon H, 3′-Geranyl-3-prenyl-5,7,2′,4′-tetrahydroxyflavone, Morusinol, Mulberrin, Moracin P, Kuwanon E, and Kuwanon A significantly inhibited the formation of cancer cell colonies. In contrast, Eleutheroside A and Morusin exhibited a partial inhibitory effect on colony formation. These effects were consistent across the two gastric cancer cell lines, MKN45 and HGC27, thereby reinforcing the potential therapeutic implications of these compounds (Figure 1D,E).

### 2.4. Multifaceted Anti-Cancer Activity: IC_50_ Measurements across Multiple Cell Lines

**Figure 2:**
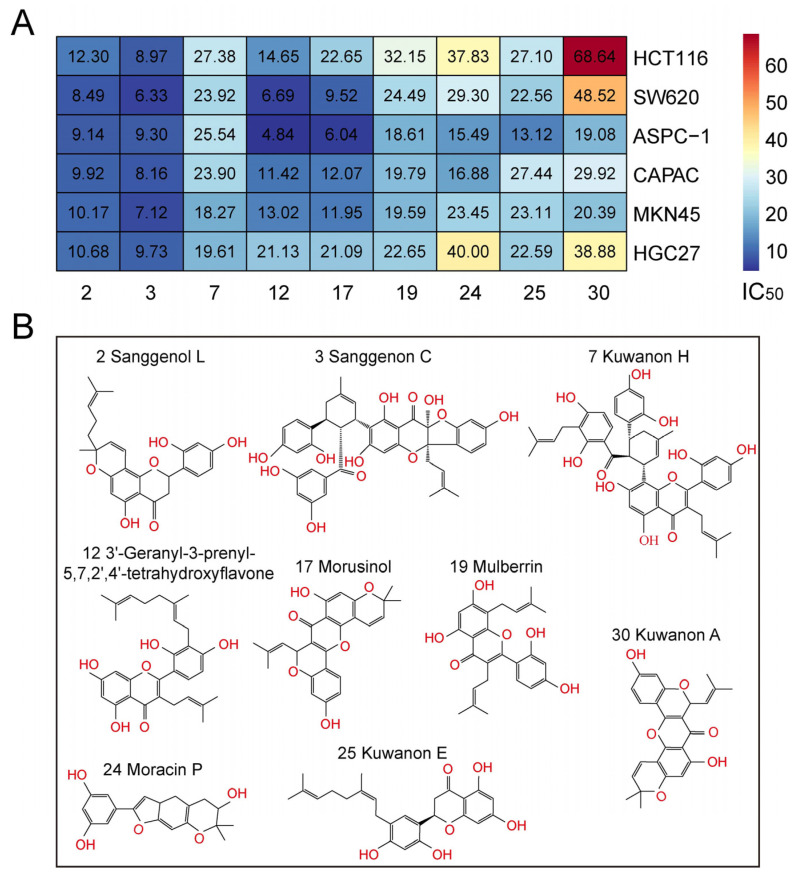
**IC_50_ values of the nine identified anti-cancer bioactive compounds from *Morus alba* L.** (**A**) Three different types of cancer: colon (HCT116 and SW620), pancreatic (ASPC-1 and CAPAN-1), and gastric (MKN45 and HGC27) were incubated with a series of concentrations (1, 2, 4, 8, 16, 32, 64, and 128 μM) of each anti-cancer bioactive compound identified in this study for 2 days. Subsequently, the IC_50_ values were calculated. (**B**) The chemical structure of the anti-cancer bioactive compound identified in this study.

A more in-depth assessment of the selected nine compounds was conducted, measuring their half-maximal inhibitory concentration (IC_50_) across six different cancer cell lines—two each from colon, pancreatic, and gastric cancers. This was performed using the MTT assay, a colorimetric assay for assessing cell metabolic activity. Overall, all the compounds demonstrated substantial anti-cancer activity, albeit with different efficacies. Notably, Sanggenol L and Sanggenon C emerged as the most potent inhibitors across all cell lines, registering low IC_50_ values of approximately 10 μM. On the other hand, 3′-Geranyl-3-prenyl-5,7,2′,4′-tetrahydroxyflavone and Morusinol exhibited significantly lower IC_50_ values for the SW620 and ASPC-1 cell lines, although their inhibitory capacities on other cell lines were slightly subdued. The remaining compounds—Kuwanon H, Moracin P, Kuwanon E, and Kuwanon A—although showing anti-cancer activity, had comparatively higher IC_50_ values, falling in the range of 20–70 μM across the different cell lines. These variances in IC_50_ values among the compounds highlight the diverse growth inhibitory capacities of these *Morus alba* L. compounds, further validating their potential therapeutic implications in different types of digestive tract cancers (Figure 2A).

These compounds predominantly fall under three major chemical classes—phenolic compounds, flavonoids, and stilbenoids. The phenolic compounds Sanggenon C and Moracin P are characterized by aromatic rings and hydroxyl groups. Flavonoids, represented by 3′-Geranyl-3-prenyl-5,7,2′,4′-tetrahydroxyflavone, exhibit a 15-carbon skeleton, typical of this class. The stilbenoids, such as Sanggenol L and Kuwanon H, possess two phenyl rings connected by a methylene bridge. Despite the differences in their individual chemical structures, these compounds share some common characteristics, including being derived from *Morus alba* L. and exhibiting notable anti-cancer activity. Furthermore, they are all organic compounds containing aromatic rings, which suggests the potential involvement of π-π stacking interactions in their mechanisms of action. Additionally, their hydroxyl functional groups could contribute to their bioactivity, possibly via hydrogen bonding or the enhancement of their solubility for improved bioavailability. Their shared biological activity against cancer cell lines underlines their therapeutic potential in the treatment of digestive tract cancers (Figure 2B).

### 2.5. The Impact of Morus alba L. Bioactive Compounds on Cell Proliferation

**Figure 3:**
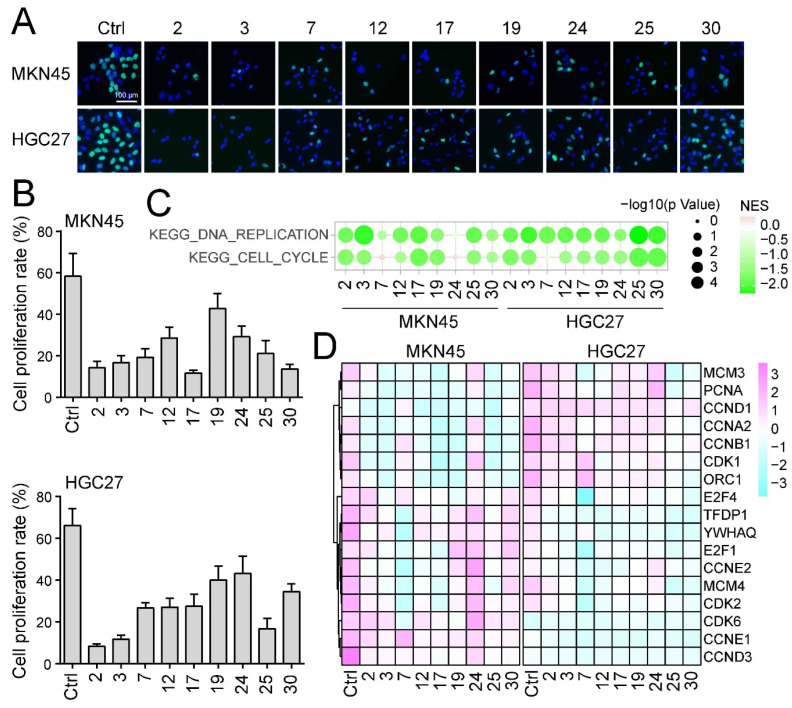
**The anti-cancer bioactive compounds demonstrate anti-cancer activity by inhibiting cell proliferation.** (**A**) Each of the nine chemical components was incubated with MKN45 and HGC27 cells for 2 days. The cell proliferation ability was subsequently evaluated using the EdU incorporation assay. The green signal represents the EdU signal, while blue indicates the nuclei. Scale bar = 100 μm. (**B**) The cell proliferation rate was statistically analyzed. For each data point, six random views were chosen to calculate the proportion of EdU-positive cells. (**C**) Gene set enrichment analysis (GSEA) was utilized to explore the alterations in the pathways associated with DNA replication and cell cycle. The size of the dots indicates the *p*-value, while the color of the dots represents the NES value. (**D**) Heatmap of the genes related to cell cycle and DNA replication.

To further elucidate the potential anti-cancer mechanisms underlying the nine selected compounds, a series of experiments were conducted to evaluate changes in cell proliferation and gene expression. The EDU cell proliferation assay, which enables the direct measurement of active DNA synthesis, revealed significant reductions in EDU signals following treatment with the selected compounds. A significant reduction in both the proportion of EDU-positive cells and the fluorescence intensity was observed in MKN45 and HGC27 cells compared to the control groups (Figure 3A,B).

Next, bulk RNA sequencing data were analyzed using gene set enrichment analysis (GSEA), revealing profound alterations in gene expression profiles post-treatment. In cells treated with Sanggenol L, Sanggenon C, 3′-Geranyl-3-prenyl-5,7,2′,4′-tetrahydroxyflavone, Morusinol, Mulberrin, Kuwanon E, or Kuwanon A, there was a significant downregulation of pathways related to cell cycle and DNA replication compared to control groups (NES > 1.5, *p* value < 0.05). However, these pathways were not significantly altered in cells treated with Kuwanon H or Moracin P (Figure 3C). Further analysis of critical genes related to DNA replication and cell cycle, including MCMs, CDKs, and cyclins, revealed marked downregulation across the board (Figure 3D). These results indicate that *Morus alba* L. monomers may exert their anti-cancer effects by inhibiting cell proliferation and disrupting normal cell cycle progression, suggesting a potential therapeutic strategy for the treatment of gastrointestinal cancer.

### 2.6. Morus alba L. Bioactive Compounds Induce ER Stress

**Figure 4:**
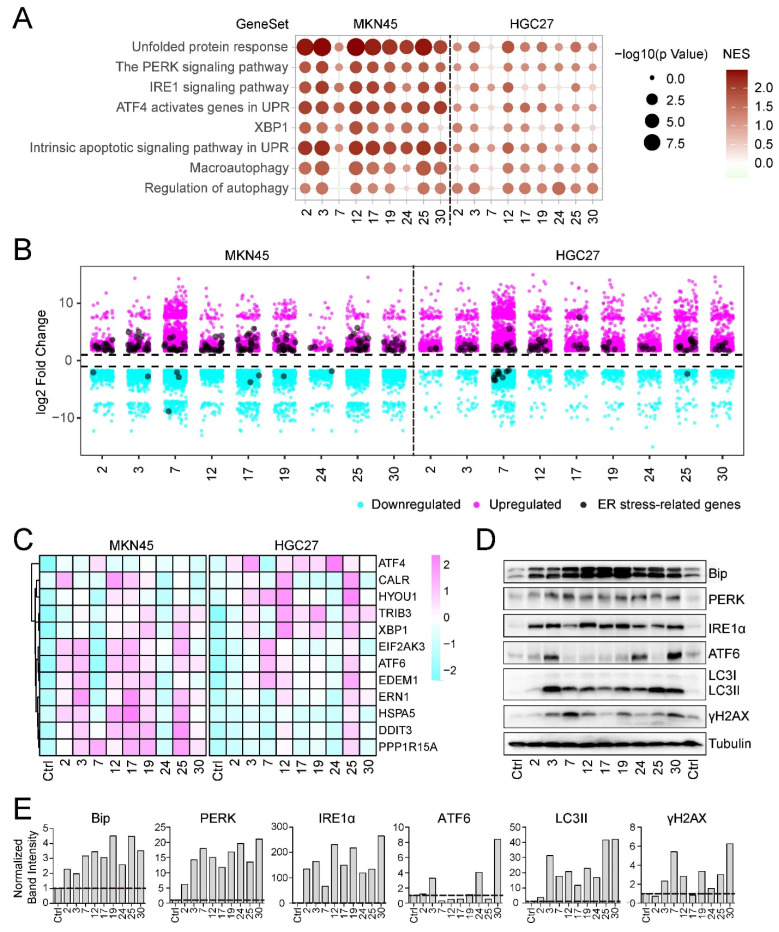
**Anti-cancer bioactive compounds induce ER stress in gastric cancer cells.** (**A**) GSEA was utilized to explore changes in ER stress, the unfolded protein response, and their related downstream pathways. The size of the dots represents the *p*-value, while the color signifies the NES value. (**B**) The volcano plot represents differentially expressed genes after cell incubation with each of the chemicals identified in this study. Orange-red and sky-blue dots represent up and downregulated genes, respectively. Black dots denote key genes related to ER stress. (**C**) The heatmap of the genes associated with the unfolded protein response and ER stress. (**D**) Western blotting was used to examine the expression of Bip, PERK, IRE1α, ATF6, LC3, and γH2AX, with tubulin used as an internal control. (**E**) Statistical analysis for the qualification of Western blotting in Panel (**D**).

A comprehensive analysis of the transcriptome data revealed that the nine bioactive compounds from *Morus alba* L. significantly induced the unfolded protein response (UPR) and endoplasmic reticulum (ER) stress. Gene set enrichment analysis (GSEA) showed that eight of these compounds—Sanggenol L, Sanggenon C, 3′-Geranyl-3-prenyl-5,7,2′,4′-tetrahydroxyflavone, Morusinol, Mulberrin, Moracin P, Kuwanon E, and Kuwanon A—triggered a marked upregulation of the UPR and ER stress. Associated pathways such as Protein kinase RNA-like Endoplasmic Reticulum Kinase (PERK), Inositol-Requiring Enzyme 1 alpha (IRE1α), and Activating Transcription Factor 4 (ATF4) were significantly upregulated upon treatment with these compounds. However, although Kuwanon H also demonstrated a similar trend, the change was relatively minor, especially in HGC27 cells (Figure 4A).

A detailed analysis of the ER stress response was accomplished using volcano plots and heatmaps. A suite of crucial ER stress-associated genes, including TRIB3, DDIT3, ATF4, and ERN1, was observed to be distinctly upregulated in the treatment groups with *Morus alba* L. bioactive compounds (Figure 4B,C). These genes are known to play pivotal roles in ER stress: TRIB3 acts as a stress-responsive gene, DDIT3 (also known as CHOP) is a crucial component of ER stress-mediated apoptosis, and ATF4 is involved in the transcriptional activation of stress response genes. Furthermore, the ER stress chaperone Bip, a critical player in initiating the unfolded protein response (UPR), was significantly upregulated upon treatment, as demonstrated by Western blot analysis (Figure 4D,E). This process is essential for the restoration of ER homeostasis by reducing the load of unfolded proteins. In addition, ER stress sensors PERK and IRE1α, which control UPR pathways, exhibited notable upregulation (Figure 4D,E), indicating activation of ER stress response. PERK signaling is critical for attenuating protein translation, whereas IRE1α is responsible for XBP1 mRNA splicing, a key player in ER stress adaptation. Lastly, substantial upregulation was observed for ATF6 following treatment with Sanggenon C, Moracin P, and Kuwanon A. ATF6 is a major ER stress transducer that induces UPR target genes to alleviate ER stress (Figure 4D,E), supporting the notion of an activated ER stress response. These findings underline the potential of *Morus alba* L. bioactive compounds in inducing ER stress and UPR as part of their anti-cancer activities.

### 2.7. Induction of Intrinsic Apoptotic Signaling and Autophagy by Morus alba L. Monomeric Compounds

In addition to ER stress, the intrinsic apoptotic signaling pathway was significantly upregulated by the *Morus alba* L. bioactive compounds (Figure 4A), highlighting another key aspect of their anti-cancer activities. The intrinsic apoptotic pathway, also known as the mitochondrial pathway, is a crucial mechanism that the cell uses to eliminate damaged cells, and it plays a critical role in response to a variety of stressors, including DNA damage, ER stress, and others. Furthermore, macroautophagy and the regulation of autophagy were significantly upregulated in response to the treatment, excluding the Kuwanon H treatment group (Figure 4A). Autophagy is a cellular degradation pathway that removes damaged proteins and organelles to maintain cellular homeostasis. It is activated in response to a range of stressors, including nutrient deprivation, hypoxia, and ER stress, and plays a dual role in cancer by inhibiting tumorigenesis and aiding cancer cell survival under stress conditions.

Western blotting results demonstrated a significant upregulation of LC3B (Figure 4D,E), a critical protein involved in autophagy and a widely used marker of autophagosome formation. This indicates that *Morus alba* L. bioactive compounds activate autophagy in cancer cells. The DNA damage marker γH2AX also showed upregulation in response to multiple compounds, including Sanggenon C, Kuwanon H, 3′-Geranyl-3-prenyl-5,7,2′,4′-tetrahydroxyflavone, Mulberrin, and Kuwanon A (Figure 4D,E). γH2AX is a variant of the H2A histone family and is a sensitive indicator of DNA double-strand breaks, suggesting that these compounds induce DNA damage in cancer cells.

In summary, these findings provide evidence that the *Morus alba* L. bioactive compounds may exert their anti-cancer effects via the induction of ER stress, activation of the intrinsic apoptotic pathway, upregulation of autophagy, and induction of DNA damage.

### 2.8. Moracin P Induces Endoplasmic Reticulum Stress and Triggers Apoptosis in Alimentary Cancer Cells

**Figure 5:**
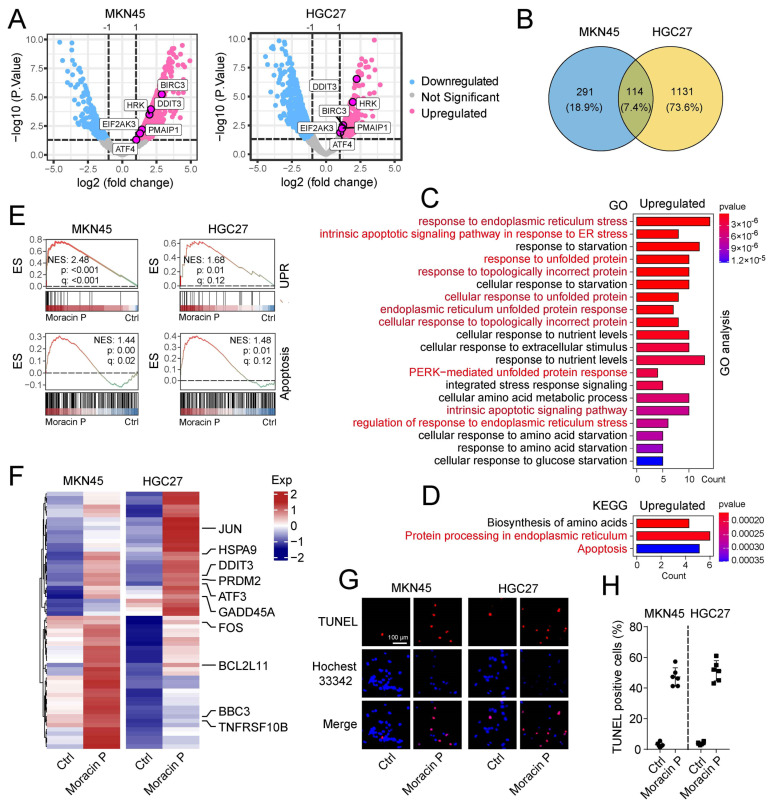
**Moracin P induces ER stress and increases apoptosis in gastric cancer cells.** (**A**) Volcano plot of differentially expressed genes post Moracin P treatment, compared to cells incubated with an equal volume of DMSO. Red and blue dots signify up and downregulated genes, respectively. Five crucial genes in the ER stress response, including DDIT3, HRK, BIRC3, ATF4, and EIF2AK3, are specifically magnified. (**B**) Venn diagram showing overlap of upregulated genes in MKN45 and HGC27 cells. GO (**C**) and KEGG (**D**) analyses of genes upregulated in both MKN45 and HGC27 cells. The red font represents the signal pathways related to ER stress and apoptosis. (**E**) GSEA of pathways related to the unfolded protein response and apoptosis. NES, *p*, and q values are labeled in each panel. (**F**) Heatmap of apoptosis-related genes after cell incubation with Moracin P, using DMSO as a control, in MKN45 and HGC27 cells. (**G**) TUNEL analysis of cells post Moracin P treatment, with DMSO serving as a negative control. Scale bar = 100 μm. The statistical analysis is presented in (**H**).

Moracin P, a bioactive compound from *Morus alba* L., shows a profound influence on gene expression in the gastric cancer cell lines MKN45 and HGC27. Following treatment with Moracin P, endoplasmic reticulum (ER) stress genes such as BIRC3, HRK, DDIT3, EIF2AK3, PMAIP1, and ATF4 (known as genes involved in ER stress response) were significantly upregulated in both cell lines (Figure 5A). Further gene expression analysis reveals that Moracin P treatment resulted in the upregulation of 405 and 1245 genes in MKN45 and HGC27 cells, respectively. Importantly, 114 genes were consistently upregulated across both cell lines (Figure 5B). Gene Ontology (GO) analysis of these 114 shared genes showed enrichment in pathways related to ER stress responses, including response to ER stress, intrinsic apoptotic signaling pathway in response to ER stress, response to unfolded protein, and PERK-mediated unfolded protein response (Figure 5C). KEGG pathway analysis of these upregulated genes revealed enrichment in protein processing in the endoplasmic reticulum and apoptosis pathways (Figure 5D). Gene set enrichment analysis (GSEA) and gene set variation analysis (GSVA) further validated these results, demonstrating that Moracin P effectively activates both the unfolded protein response and apoptosis pathways in MKN45 and HGC27 cells (Figure 5E and Figure S1). Particularly noteworthy is the upregulation of key apoptosis pathway genes, such as DDIT3 (also known as CHOP), BCL2L11 (Bim), BBC3 (PUMA), and GADD45A, which are known to modulate cell death processes (Figure 5F). TUNEL assay, a common method for detecting DNA fragmentation that results from apoptotic signaling cascades, further substantiated these findings. Upon Moracin P treatment, a significant increase in TUNEL-positive signals was observed (Figure 5G,H), indicating a substantial induction of apoptosis in the treated cells. In summary, these findings clearly illustrate the ability of Moracin P to induce potent ER stress responses, which leads to the upregulation of apoptotic signaling pathways and ultimately results in cell death. The evident induction of apoptosis further supports Moracin P’s potential as a therapeutic agent for gastric cancer.

### 2.9. In Vivo Anti-Cancer Efficacy of Moracin P in Mouse Models of Gastrointestinal Cancer

**Figure 6:**
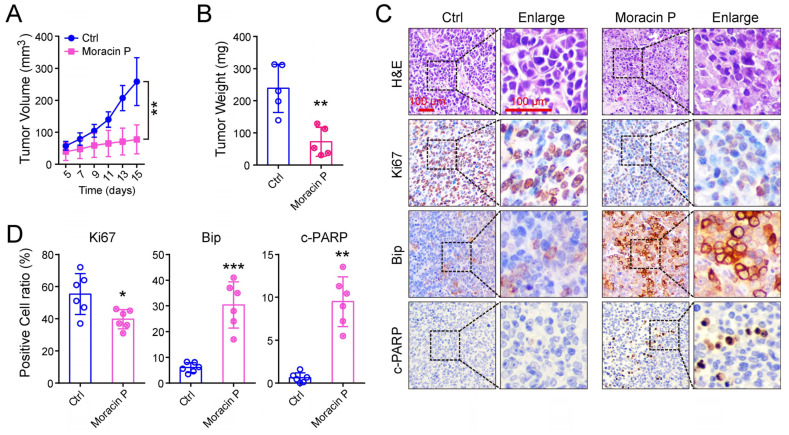
**Moracin P inhibits gastric cancer cell growth, induces the ER stress response, and triggers apoptosis in vivo.** (**A**) Subcutaneous xenograft mouse models were established using MKN45 cells, followed by treatment with either Moracin P or DMSO. The panel also illustrates tumor growth curves. (**B**) The graph shows tumor weight. (**C**) Hematoxylin and eosin (HE), Ki67, Bip, and c-PARP staining of tumor tissues are displayed. Scale bar = 100 μm. (**D**) The statistical analysis depicts the ratio of positive cells for Ki67, Bip, and c-PARP. Data are presented as mean ± SD; * *p* < 0.05, ** *p* < 0.01, *** *p* < 0.001, in comparison with the control group.

An in vivo assessment of Moracin P’s anti-cancer properties was undertaken using a mouse model. Tumor growth in the Moracin P group was notably smaller than in the control group, with significant reductions in both tumor volume and weight. Histological examination (H&E staining) revealed that the cell density in the Moracin P treatment group was noticeably lower, indicating a reduction in tumor cell proliferation. This could potentially be interpreted as Moracin P ameliorating the malignancy of the tumor (Figure S2 and Figure 6A,B). Immunohistochemistry results demonstrated that Ki67, an indicator of cell proliferation widely utilized in cancer research and diagnostics, was notably diminished. In contrast, Bip, indicative of ER stress, and cleaved PARP, a hallmark of apoptosis, both showed marked upregulation in the group treated with Moracin P (Figure 6C,D). These results suggest that Moracin P has promising anti-cancer potential, significantly inhibiting tumor growth and inducing cell apoptosis in vivo.

### 2.10. Potential Anti-Cancer Mechanisms of Moracin P Revealed by Target-Based Enrichment Analysis

**Figure 7:**
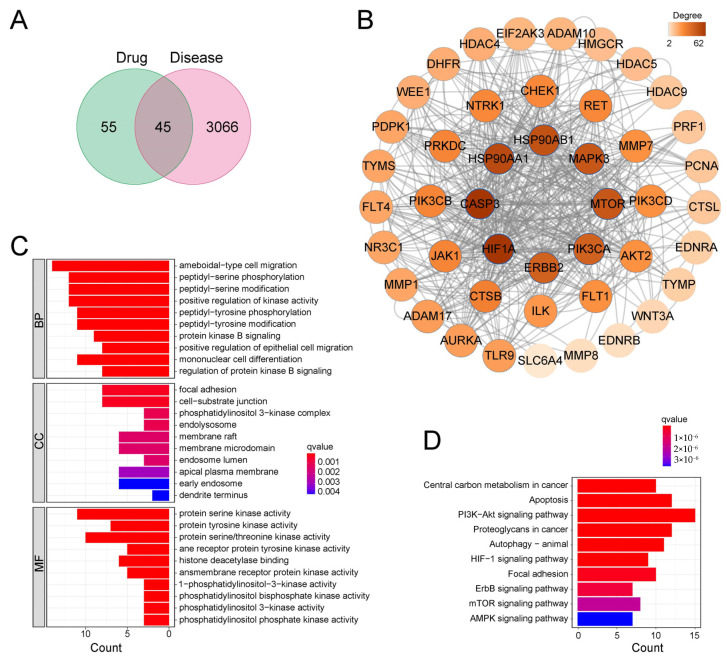
**Target-based enrichment analysis of Moracin P against gastric cancer cells.** (**A**) Venn diagram representing potential targets of Moracin P specific to gastric cancer. “Drug” represents the identified 100 targets of Moracin P from the Swiss Target Prediction, PharmMapper, and SEA databases, and “Disease” represents the gastric cancer-related targets sourced from the GeneCard, OMIM, and PharmGKB databases. (**B**) Protein–Protein Interaction (PPI) network analysis involving 45 targets of Moracin P. histograms depicting gene ontology (GO). (**C**) and Kyoto Encyclopedia of Genes and Genomes (KEGG). (**D**) Enrichment analysis of targets.

To further determine the potential anti-cancer mechanisms of Moracin P against gastric cancer, a target-based enrichment analysis was conducted. Initially, 100 targets of Moracin P were identified from the Swiss Target Prediction, PharmMapper, and SEA databases. Among these, 45 targets were found to overlap with gastric cancer-related targets sourced from the GeneCard, OMIM, and PharmGKB databases (Figure 7A). Subsequently, a PPI network was constructed around these intersecting targets, comprising 45 nodes and 522 edges (Figure 7B). Within this network, the top eight targets with the highest degrees were HIF1A, CASP3, HSP90AA1, HSP90AB1, MAPK8, MTOR, PIK3CA, and ERBB2, highlighting their pivotal roles. Enrichment analysis using GO and KEGG was then applied to these shared targets (Figure 7C,D). The GO enrichment analysis suggested that Moracin P might inhibit serine and tyrosine kinase activity in gastric cancer cells (Figure 7C). Concurrently, the KEGG pathway enrichment analysis revealed a significant association of Moracin P targets with central carbon metabolism, apoptosis, autophagy, and proteoglycan pathways (Figure 7D).

## 3. Discussion

Natural products, predominantly derived from plants, have played a pivotal role in therapeutic interventions, significantly contributing to the discovery of new drugs for a wide range of diseases [108,109], including cancer [110], infectious diseases [111], cardiovascular diseases [112], and neurological disorders [113]. Plant-derived compounds, such as irinotecan, vincristine, etoposide, and paclitaxel [114], have demonstrated efficacy against various cancers, significantly improving both survival rates and the quality of life for millions of cancer patients. The current landscape of natural products in cancer therapy remains promising, with a substantial proportion of anti-tumor drugs either originating from natural products or inspired by them. Furthermore, as our understanding of cancer biology deepens, the multifaceted mechanisms by which many natural compounds operate are becoming apparent. These compounds not only exhibit cytotoxicity against tumor cells but also modulate the tumor microenvironment, making them ideal candidates for combinatorial therapies [115]. While the past is filled with success stories of natural products, the present is marked by technological advancements that enable systematic study, extraction, and synthesis of these compounds. The synergy of traditional knowledge with modern science ensures a bright future for the role of natural products in cancer therapeutics.

*Morus alba* L., commonly known as white mulberry, has a rich history in traditional medicine systems. Various parts of this plant, including the roots, leaves, and fruits, have been utilized for a wide range of therapeutic applications. Recent scientific investigations have unveiled a plethora of bioactive compounds within *Morus alba* L., showcasing diverse biological activities such as anti-inflammatory, antioxidant, and anti-diabetic effects [14]. Our current research reveals the pronounced anti-cancer properties of *Morus alba* L. monomers. These compounds exhibit robust inhibitory effects on crucial mechanisms governing cancer progression, particularly the cell cycle and DNA replication-related gene expression. Furthermore, the induction of the unfolded protein response (UPR) and endoplasmic reticulum (ER) stress by these monomers presents a fascinating therapeutic dimension. This phenomenon could lead to DNA damage, autophagy, and subsequent cancer cell apoptosis, establishing these monomers as versatile agents against gastrointestinal cancers. Considering the urgent need for effective cancer therapeutics with favorable safety profiles, the role of *Morus alba* L. monomers is of paramount importance. Their botanical origin may offer improved pharmacokinetic properties and safety profiles, potentially paving the way for their future incorporation in both mono and combination cancer therapies. As the field of oncology continues to evolve, the therapeutic significance of such monomers is expected to receive increasing attention.

Our study notably reveals that *Morus alba* L. compounds induce significant ER stress and UPR in cancer cells. While the UPR typically acts as a cell’s defense mechanism, ensuring cellular protein homeostasis [116], it can also initiate cell death when the damage is too extensive [117]. Recent insights suggest leveraging this UPR activation as a cancer treatment strategy [118], given that cancer cells, already stressed, might be especially vulnerable to agents that amplify this stress. *Morus alba* L. compounds might strategically disrupt the cellular stress response pathways of cancer cells. Interestingly, when these compounds are combined with other therapeutic agents or strategies, they could enhance the effectiveness of those therapies, providing a two-pronged approach against the therapeutic resistance of cancer cells. Previous studies have shown that the combination of Kuwanon-A and 5-fluorouracil reduced tumor progression in gastric cancer via the synergistic activation of Chop, recognized as a key mediator of ER stress-induced pathways [15]. Moreover, the selectivity of *Morus alba* L. compounds is worth exploring further. Since non-transformed or healthy cells do not usually have increased ER stress like cancer cells, drugs that intensify ER stress might have a wide range of effective doses. This means they could target and kill cancer cells while leaving healthy cells unaffected.

Research has demonstrated that certain components derived from *Morus alba* L. exhibit anti-cancer activity and are involved in ER stress and its related pathways in cancer cells. Polyphenol extracts from mulberry leaves can counteract the resistance to doxorubicin induced by ER stress, showing potential inhibitory effects on HepG2 hepatoma cells and suggesting anti-cancer properties linked to ER stress modulation [119]. Kuwanon M, also derived from *Morus alba*, triggers both apoptosis and paraptosis in cancer cells by activating ER stress and the UPR response [120]. Another compound from *Morus alba*, Kuwanon A, has been found to inhibit the growth of gastric cancer cells via ER-stress-mediated apoptosis [15]. Kuwanon H suppresses melanoma progression by inducing ER stress cytotoxicity and disrupting autophagy flux [40]. With these increasing findings highlighting the role of *Morus alba* L. extracts and compounds in modulating ER stress and UPR in various cancer types, it becomes evident that these compounds represent a valuable source of potential therapeutic agents. The multifaceted interactions between these compounds and ER stress pathways underscore their ability to target cancer cells at different stages and via various mechanisms.

The dynamic interplay between ER stress, UPR, and cancer progression is complex. While ER stress can promote apoptosis in some contexts, it may also support tumor growth and survival under specific conditions [121]. *Morus alba* L. compounds demonstrate a unique ability to exploit this dual nature by enhancing the pro-apoptotic effects of ER stress in cancer cells, making them promising candidates for cancer therapy. In the present study, there was a significant upregulation in the expression of CHOP and the intrinsic apoptotic signaling pathway within the UPR after treatment with these compounds. Such findings suggest that *Morus alba* L. derivatives might be eliciting a strong ER stress response, driving cancer cells toward apoptosis. This could provide a therapeutic advantage by targeting the vulnerabilities of cancer cells, thereby undermining their adaptive survival mechanisms. Further investigations into the specific interactions of these compounds with CHOP and the intrinsic apoptotic pathway can pave the way for refining and potentially optimizing their anti-cancer efficacy.

PERK, IRE1α, and ATF6 are critical mediators of the unfolded protein response (UPR) in endoplasmic reticulum (ER) stress, influencing cancer progression, survival, and therapy resistance by modulating cellular responses ranging from adaptive survival to apoptosis [122]. The differential induction patterns of PERK, IRE1alpha, and ATF6 by the detected monomers underscore the varied mechanisms through which *Morus alba* L. compounds may exert their effects. While all detected monomers activated PERK and IRE1α, only Sanggenon C, Moracin P, and Kuwanon A upregulated ATF6 expression. This observation is particularly intriguing, considering the distinct roles each of these sensors play in the UPR. PERK and IRE1α activation are common cellular responses to mitigate ER stress by attenuating global protein synthesis and increasing the degradation of misfolded proteins, respectively. Their universal upregulation by all tested monomers suggests a broad impact on ER stress modulation by *Morus alba* L. compounds. In contrast, ATF6, functioning as a transcriptional activator under ER stress conditions, was only induced by specific monomers. Its selective upregulation might indicate specialized functions or therapeutic potentials for Sanggenon C, Moracin P, and Kuwanon A. The non-induction of ATF6 by other monomers raises questions about their specific modes of action and whether they act more predominantly via the PERK and IRE1α pathways or other as-yet-unidentified mechanisms. Further investigations into the distinct molecular interactions and signaling outcomes driven by each monomer will be crucial. Examining these pathways will lead to a more thorough understanding of their potential therapeutic implications and the optimization of treatment regimens utilizing these compounds.

ER stress can trigger multiple cell death mechanisms, including apoptosis, autophagy, necrosis, pyroptosis, and ferroptosis. Focusing on these mechanisms, particularly the interactions among ER stress, apoptosis, and autophagy, offers promising therapeutic avenues [123]. The detected monomers notably activate autophagy, as evidenced by the upregulation of LC3B, particularly LC3II—a hallmark of autophagosome formation—and the activation of the macroautophagy pathway, highlighting its crucial role in autophagy regulation. The balance between ER stress and autophagy is particularly intriguing in the context of cancer therapeutics. While autophagy traditionally acts as a survival mechanism under conditions of nutrient deprivation or cellular stress, its activation or autophagic flux impairment can also lead to cell death, especially when combined with other stressors like ER stress. This dual nature of autophagy, as both a pro-survival and a pro-death mechanism, largely depends on the cellular context and the nature and duration of the inducing signals. The role of autophagy induced by these monomers is still under investigation, but the induction of DNA damage suggests that this autophagy may be leading to cell death. Although the precise role of autophagy in the context of these monomers remains a subject of ongoing research, preliminary findings pointing to DNA damage and cell death are promising. Future studies should prioritize the mechanistic understanding of these processes, paving the way for potential clinical applications of these compounds in cancer therapy.

Among the tested compounds, Moracin P stood out due to its potent induction of ER stress responses and apoptosis. The in vivo evidence further bolstered Moracin P’s potential as a therapeutic agent. It is important to note, however, that while our findings present a promising step toward harnessing *Morus alba* L. compounds for cancer therapy, additional in-depth studies are necessary. Future research should aim to elucidate the precise molecular targets of these compounds and evaluate their efficacy in clinical trials.

Indeed, while the present study provides a promising foundation for understanding the anti-cancer potential of *Morus alba* L. compounds, there are specific areas that require further research. Firstly, this study conducted in vivo validation for Moracin P, yielding encouraging results in reducing tumor growth. However, in vivo investigations for other bioactive compounds are necessary. This would not only confirm the in vitro findings but also offer a more comprehensive assessment of each compound’s anti-cancer potential. Secondly, although these compounds have shown potent anti-cancer activity, their safety profiles need rigorous evaluation. Future research should, therefore, include a thorough assessment of the toxicity of these compounds, both individually and when administered in combination. This is crucial for establishing a therapeutic window and preventing potential adverse effects during treatment. Additionally, a comprehensive characterization of the pharmacokinetic properties of these compounds is warranted. This involves determining their absorption, distribution, metabolism, and excretion profiles. Such studies could provide insights into the bioavailability of these compounds and their stability in biological systems. Consequently, this knowledge could guide the design of suitable drug delivery systems to enhance their therapeutic efficacy. Collectively, these future research directions are vital for translating the present findings into clinical applications. Achieving this will require multidisciplinary collaborations involving oncologists, pharmacologists, and medicinal chemists, highlighting the complexity and interdisciplinary nature of cancer research. Overall, while the journey from bench to bedside is often challenging and lengthy, the findings of this study offer a promising starting point for developing *Morus alba* L. compounds as potential therapeutics for gastrointestinal cancers.

## 4. Materials and Methods

### 4.1. Cell Culture and Reagents

The human colon cancer cell line HCT116 was cultured in McCoy’s 5A medium, while SW620 colon cancer cells, ASPC-1 and CAPAN-1 pancreatic cancer cells, and MKN 45 and HGC 27 gastric cancer cells were cultured in DMEM medium. All media were supplemented with 10% fetal bovine serum (FBS, Gibco, Grand Island, NY, USA), and the cells were incubated at 37 °C in a 5% CO_2_ incubator. The mulberry extractions listed in Table 1 were provided by Chengdu DeSiTe Biological Technology Co., Ltd., Chengdu, China, with a purity greater than 98%. MTT (Methylthiazolyldiphenyl-tetrazolium bromide, ST316), One Step TUNEL Apoptosis Assay Kit (C1090), BeyoClick™ EdU Cell Proliferation Kit with Alexa Fluor 488 (C0071S), and BeyoECL Star (P0018AM) were purchased from Beyotime (Beijing, China). Antibodies for Bip (#3177), PERK (#5683), IRE1α (#3294), ATF6 (#65880), LC3 (#4599), γ-H2AX (#60566), Tubulin (#2146), Ki67 (#34330), and Cleaved-PARP (#5625) were purchased from Cell Signaling Technology (Danvers, MA, USA). The SuperSignal WestFemto Maximum Sensitivity Substrate was provided by Thermo Scientific (Carlsbad, CA, USA).

### 4.2. Crystal Violet Staining

The test cells (MKN 45 and HGC 27) were seeded into 24-well plates at a density of 1 × 10^3^ cells/well. Once the cells adhered to the plates, they were treated with various drugs at a concentration of 100 μM and maintained at 37 °C in a 5% CO_2_ incubator. After a period of 2–3 weeks, the cells were fixed with 3.7% paraformaldehyde (PFA) for 15 min and subsequently stained with Crystal Violet Staining Solution (C0121, Beyotime, Beijing, China). The plates were then rinsed with fresh phosphate-buffered saline (PBS) buffer and imaged using a scanner (Epson, Los Alamitos, CA, USA).

### 4.3. MTT Assay

The human colon cancer cell lines HCT116 and SW620, along with ASPC-1 and CAPAN-1 pancreatic cancer cells, as well as MKN45 and HGC27 gastric cancer cells, were seeded into 96-well plates at a density of 1 × 10^3^ cells per well. They were then treated with varying concentrations (1, 2, 4, 8, 16, 32, 64, and 128 μM) of the monomers listed in Table 1. Seventy-two hours post-treatment, the cells were incubated with MTT solution at 37 °C for 2 h. The supernatant was then discarded, and DMSO (100 μL per well) was added to fully dissolve the MTT formazan crystals. The plates were subsequently placed in a multifunctional microplate reader to measure the absorbance at 492 nm.

### 4.4. Bulk RNA-Seq and Data Analysis

The experimental cells were exposed to the preselected drugs at the IC_50_ concentration for two days. Following this, the cells were collected and immediately lysed in RNAiso Plus (#9108, Takara Bio Inc., Shiga, Japan). The total RNA was then extracted using the classical phenol–chloroform extraction method, strictly according to the operation manual. The quantification and qualification of RNA, the preparation of the library for transcriptome sequencing, and quality control and comparative analysis were all conducted at Biomarker Technologies in Beijing, China. The bulk RNA-seq data were analyzed using the R programming language. The gene name conversion was accomplished using the org.Hs.eg.db (v.3.16.0) package, while the differential gene expression was analyzed using the edgeR (v.3.40.2) package. The gene set enrichment analysis (GSEA) was executed with the fgsea (v.1.24.0) package and visualized using either the ggplot2 (v.3.4.2) or GseaVis (v.0.0.8) packages. Gene set variation analysis (GSVA) was conducted using the GSVA (v.1.46.0) package. The gene expression heatmap was generated using the pheatmap (v.1.0.12) package. Kyoto Encyclopedia of Genes and Genomes (KEGG) and Gene Ontology (GO) analyses were performed with the aid of the clusterProfiler (v.4.7.1.2) and enrichplot (v.1.18.4) packages.

### 4.5. TUNEL Assay

The TUNEL assay was conducted using the One Step TUNEL Apoptosis Assay Kit according to the guidelines provided in the user manual. In summary, the process begins with collecting the free-floating cells from the culture medium and subjecting the adherent cells to enzymatic disintegration to achieve a single-cell suspension. Subsequently, a centrifugation process is undertaken at 700 rpm, 4 °C for five minutes, with the supernatant being discarded thereafter. The cell pellet is promptly resuspended in 3.7% PFA for a 30 min fixation at ambient temperature. Once washed, the cells are gently resuspended in PBS that contains 0.3% Triton X-100, followed by an incubation at room temperature for five minutes. After a further PBS wash, the cells are exposed to a freshly concocted TUNEL detection solution at 37 °C, shielded from light, for 60 min. In the final step, the cell nuclei are stained with Hoechst 33342. The concentrated cell suspension is then applied onto a glass slide, covered with a coverslip, and visualized and photographed with a fluorescence microscope.

### 4.6. Western Blotting

Western blotting was conducted as described previously [124,125]. Briefly, fifty micrograms of protein from each sample were individually segregated using SDS-PAGE gel and subsequently transferred onto a PVDF membrane via a wet transfer method according to the methods described in our previous studies. Subsequently, the membrane was blocked with 5% BSA in TBST buffer, followed by incubation with primary antibodies against Bip, PERK, IRE1α, ATF6, LC3, γ-H2AX, and Tubulin at dilutions of 1:1000 for all except Tubulin, which was at 1:5000. This was left to incubate at 4 °C overnight. After a series of washes with fresh TBST buffer, the membrane was exposed to horseradish peroxidase (HRP)-conjugated secondary antibodies for 2 h at room temperature. Lastly, the target bands were made visible with the SuperSignal WestFemto Maximum Sensitivity Substrate, using a Western blot detection instrument from Clinx Science (Shanghai, China).

### 4.7. EdU Incorporation Assay

The EdU (5-Ethynyl-2′-deoxyuridine) incorporation assay was performed using the BeyoClick™ EdU Cell Proliferation Kit with Alexa Fluor 488 (Beyotime, China), following the instructions provided in the operating manual. Briefly, the cells were seeded into 24-well plates at a density of 1 × 10^4^ cells per well. Once the cells adhered to the plates, they were treated with various drugs and maintained at 37 °C in a 5% CO_2_ incubator for 2 days. Then, the test cells were incubated with 10 μM of EdU for 1 h. Subsequently, the cells were fixed with 3.7% paraformaldehyde (PFA) for 15 min, followed by washing in PBS and permeabilization with 0.3% Triton X-100. The cells were then stained with freshly made Click Additive Solution at room temperature for 30 min. Nuclei were counterstained with DAPI (2-(4-Amidinophenyl)-6-indolecarbamidine dihydrochloride, Beyotime, China). Finally, images were captured using a fluorescence microscope.

### 4.8. Hematoxylin-Eosin (H&E) Staining

The H&E staining was conducted as described previously [126,127]. Briefly, after being fixed with 3.7% paraformaldehyde (PFA), xenograft tumors were embedded in paraffin and sliced into 5 μm sections using an Ultra-Thin Semiautomatic Microtome (RM22, Leica, Wetzlar, Germany). The sections were deparaffinized, rehydrated, and subsequently stained with hematoxylin and eosin (H&E) using standard protocols (ZSGB-BIO, Beijing, China). Finally, the H&E-stained sections were captured under a light microscope.

### 4.9. Immunohistochemistry

The paraffin-embedded sections were subjected to heating at 65 °C to melt the paraffin. These sections were then deparaffinized, followed by a process of rehydration using a series of ethanol solutions in descending concentrations. Subsequently, antigen retrieval was performed by treating the sections with an Antigen Unmasking Solution (Citric Acid Based, Vector laboratories, Newark, CA, USA, H-3300). Sections underwent a blocking process and were subsequently incubated overnight at 4 °C with primary antibodies. This was followed by a washing step and incubation with HRP-conjugated secondary antibodies for a duration of 30 min. Visualization of the targets was facilitated by the application of diaminobenzidine (DAB, ZLI-9033, ZSGB-BIO, Beijing, China). The final phase of the procedure involved staining the slides with hematoxylin (ZSGB-BIO, Beijing, China), post which the images were captured under a microscope for further analysis.

### 4.10. Toxicity Prediction

Toxicological assessment is essential to evaluate the viability of a drug during development. In this research’s methodology, toxicity predictions for examined compounds were conducted using the ProTox-II platform (ProTox-II—Prediction of TOXicity of Chemicals, https://tox-new.charite.de/protox_II/, accessed on 10 July 2023). This platform is instrumental in the forecast and evaluation of chemical toxicity. Initially, the Simplified Molecular-Input Line-Entry System (SMILES) notations of the compounds were retrieved from PubChem, an open chemistry database (accessible at https://pubchem.ncbi.nlm.nih.gov/, accessed on 10 July 2023). These notations offer a compact textual representation of the compound structure, which is crucial for the subsequent analysis processes. Following the retrieval, the ProTox-II platform was employed to evaluate the toxicity endpoints of the compounds. The evaluation encompassed several significant endpoints, including carcinogenicity, immunotoxicity, mutagenicity, and cytotoxicity. Each of these endpoints provides essential insights into the potential adverse effects and hazardous characteristics exhibited by the compounds. Additionally, the platform predicted the Median Lethal Dose (LD50), expressed in mg/kg, which is crucial for assessing the compounds’ acute toxicity. The LD50 values were subsequently classified into the following categories: Class 1 signifies that the compound is fatal if swallowed with an LD50 value of 5 or less; Class 2, also fatal if swallowed, encompasses LD50 values between 5 and 50; Class 3, toxic if swallowed, includes LD50 values ranging from 50 to 300; Class 4 indicates that the compound is harmful if swallowed with LD50 values between 300 and 2000; Class 5, potentially harmful if swallowed, pertains to LD50 values between 2000 and 5000; and finally, Class 6, designating non-toxic compounds, refers to those with LD50 values above 5000.

### 4.11. Targets Identification and PPI Network Construction

Potential protein targets of Moracin P were sourced from online databases such as Swiss Target Prediction (http://swisstargetprediction.ch/result.php?job=924272227&organism=Homo_sapiens, accessed on 20 July 2023), PharmMapper (https://www.lilab-ecust.cn/pharmmapper/results/240111144307, accessed on 20 July 2023), and the similarity ensemble approach (SEA, https://sea.bkslab.org/jobs/search_b9291039-8c7f-4883-b53f-1d2276d5946c, accessed on 20 July 2023). After consolidating all Moracin P-related targets and eliminating duplicates, 100 unique targets for Moracin P were identified. Subsequent databases, including GeneCard (https://www.genecards.org/Search/Keyword?queryString=gastric%20cancer, accessed on 21 July 2023), OMIM (https://www.omim.org/search?index=geneMap&start=1&sort=chromosome_number+asc%2C+chromosome_sort+asc&search=gastric+cancer&limit=10, accessed on 21 July 2023), and PharmGKB (https://www.pharmgkb.org/disease/PA445742/clinicalAnnotation, accessed on 21 July 2023), were utilized to gather genes related to gastric cancer. Using the “VennDiagram” R package, Moracin P-related targets specific to gastric cancer were pinpointed. To explore the relationships among these intersecting targets, the Retrieval of Interacting Genes (STRING) database was employed. A Protein–Protein Interaction (PPI) network was constructed, setting a minimum required interaction score of 0.40. This PPI network was subsequently refined and analyzed using Cytoscape-3.9.0 software.

### 4.12. GO and KEGG Enrichment Analysis

The R package “org.Hs.eg.db” facilitated the conversion of target gene symbols to entrezID. Subsequent Gene Ontology (GO) and Kyoto Encyclopedia of Genes and Genomes (KEGG) pathway analyses were conducted using the R packages “clusterProfiler”, “org.Hs.eg.db”, “enrichplot” and “ggplot2”, with adjusted *p*-values set at <0.05.

### 4.13. Statistical Analysis

All in vitro and in vivo experiments were conducted as a minimum of three independent trials. The collected data are represented as the mean values, with the standard deviation (SD) provided for each set. The statistical analysis was performed by GraphPad Prism 6.0 (GraphPad Software Inc., San Diego, CA, USA). The unpaired Student’s *t*-test was used for comparisons between the control and experimental groups, and consideration of a *p*-value less than 0.05 signifies statistical significance. The significance levels are indicated as follows: * for *p* < 0.05, ** for *p* < 0.01, and *** for *p* < 0.001. “NS” denotes instances where differences do not reach statistical significance.

## 5. Conclusions

This study sheds light on the complex anti-cancer activities of *Morus alba* L. bioactive compounds and lays a solid foundation for their further exploration as potential therapeutic agents in the fight against cancer. Their shared ability to induce ER stress, activate intrinsic apoptotic signaling, upregulate autophagy, and induce DNA damage is of therapeutic interest and holds promise for the development of innovative anti-cancer therapies.

## Figures and Tables

**Table 1:** **Summary of bioactive compounds in *Morus alba* L. examined in this study.** CAS, catalog number; MF, molecular formula; MW, molecular weight. NA, no reports.

No.	Name	CAS	MF	MW	Functional Groups	Resource	Reported Anti-Cancer Activity
1	Mulberrofuran Q	101383-35-1	C_34_H_24_O_10_	592.5	Flavonoids	Root/bark	Against lung cancer [19]
2	Sanggenol L	329319-20-2	C_25_H_26_O_6_	422.5	Flavonoids	Root/bark	Against prostate [20] and ovarian cancer [21], as well as melanoma [21,22]
3	Sanggenon C	80651-76-9	C_40_H_36_O_12_	708.7	Flavonoids	Root/bark	Against colorectal [23], breast [24], gastric [25], and prostate cancer [26], as well as leukemia [27], and glioblastoma [17]
4	Astragalin	480-10-4	C_21_H_20_O_11_	448.4	Flavonoids	Leaf	Against lung [28,29], gastric [30,31], colorectal [32], breast [33], kidney [34], ovarian [35], liver [36], and prostate cancer [37], as well as leukemia [38] and melanoma [39]
5	Kuwanon G	75629-19-5	C_40_H_36_O_11_	692.7	Flavonoids	Root/bark/leaf	NA
6	Scopoloside	531-44-2	C_16_H_18_O_9_	354.31	Phenolic acids	Root/leaf/mulberry	NA
7	Kuwanon H	76472-87-2	C_45_H_44_O_11_	760.8	Flavonoids	Root/bark/leaf	Melanoma [40]
8	Beta-Sitosterol	83-46-5	C_29_H_50_O	414.7	Sterols	Root/bark/leaf/mulberry	Against liver, cervical, colorectal, stomach, breast, lung, pancreatic, and prostate cancer, as well as leukemia, multiple myeloma, melanoma, and fibrosarcoma [41].
9	Mulberroside A	102841-42-9	C_26_H_32_O_14_	568.5	Glycosides	Root/bark	NA
10	Oxyresveratrol 3′-O-β-D-glucopyranoside	144525-40-6	C_20_H_22_O_9_	406.4	Phenols/Glycosides	Root/leaf/mulberry	NA
11	Eleutheroside A	474-58-8	C_35_H_60_O_6_	576.8	Glycosides	Root/leaf	NA
12	3′-Geranyl-3-prenyl-5,7,2′,4′-tetrahydroxyflavone	1334309-44-2	C_30_H_34_O_6_	490.6	Flavonoids	Root/bark	NA
13	Morusin	62596-29-6	C_25_H_24_O_6_	420.5	Flavonoids	Root/bark	Against cervical [42], breast [43,44,45], colorectal [46,47], gastric [48], prostate [49,50,51], lung [52,53,54], ovarian [55], liver [56,57], and pancreatic cancer [58], as well as melanoma [59] and renal cell carcinoma [60]
14	Oxyresveratrol 2-O-β-D-glucopyranoside	392274-22-5	C_20_H_22_O_9_	406.4	Phenols/Glycosides	Root/leaf/mulberry	NA
15	1-Deoxynojirimycin	19130-96-2	C_6_H_13_NO_4_	163.17	Alkaloids	Leaf	Against colorectal [61] and breast cancer [62], as well as melanoma [63]
16	Chlorogenic acid	327-97-9	C_16_H_18_O_9_	354.31	Phenolic acids	Leaf	Against liver [64], lung [65,66], colorectal [67,68], breast [69,70,71], and kidney cancer [72], as well as glioma [65,73] and neuroblastoma [67,68,69,70,71,72,74]
17	Morusinol	62949-93-3	C_25_H_26_O_7_	438.5	Flavonoids	Root/bark	Against colorectal cancer [75] and melanoma [76]
18	Umbelliferone	93-35-6	C_9_H_6_O_3_	162.14	Ketones	Bark/leaf	Against liver [77,78], prostate [79], colorectal [80], and lung cancer [81], as well as oral carcinoma [82,83] and renal cell carcinoma [83,84]
19	Mulberrin	62949-79-5	C_25_H_26_O_6_	422.5	Glycosides	Root/bark	NA
20	Multicaulisin	286461-76-5	C_40_H_36_O_11_	692.7	Flavonoids	Root/bark	NA
21	Mulberroside C	102841-43-0	C_24_H_26_O_9_	458.5g	Glycosides	Root/bark	NA
22	Mulberroside F	193483-95-3	C_26_H_30_O_14_	566.5	Glycosides	Root/bark	NA
23	Sanggenone H	86450-80-8	C_20_H_18_O_6_	354.4	Flavonoids	Root/bark	NA
24	Moracin P	102841-46-3	C_19_H_18_O_5_	326.35	Flavonoids	Root/bark	NA
25	Kuwanon E	68401-05-8	C_25_H_28_O_6_	424.5	Flavonoids	Root/bark	NA
26	Oxyresveratrol	29700-22-9	C_14_H_12_O_4_	244.24	Phenols	Root/bark	Against colorectal [85,86], breast [87,88], cervical [89], liver [90,91], and lung cancer [92], as well as neuroblastoma [93], head and neck squamous cell carcinoma [94], and osteosarcoma [95]
27	Resveratrol	501-36-0	C_14_H_12_O_3_	228.24	Phenols	Mulberry	Against hepatic, pancreatic, post-menopausal breast, prostate, lung, and colorectal cancer, as well as hematological malignancies [96]
28	Morin	480-16-0	C_15_H_10_O_7_	302.23	Flavonoids	Bark	Against breast [97,98,99], lung [100], prostate [101], colorectal [102], ovarian [103,104], cervical [105], and bladder [106] cancer, as well as leukemia [107]
29	Moracin O	123702-97-6	C_19_H_18_O_5_	326.3	Flavonoids	Root/bark	NA
30	Kuwanon A	62949-77-3	C_25_H_24_O_6_	420.5	Flavonoids	Root/bark/leaf	Against gastric cancer [15]

## Data Availability

The datasets generated during and/or analyzed during the current study are available from the corresponding author upon reasonable request.

## Supplementary Materials

The supporting information can be downloaded at https://www.mdpi.com/article/10.3390/ijms25020999/s1.
