# Supplementary material for: Gastrointestinal Cancer Therapeutics via Triggering Unfolded Protein Response and Endoplasmic Reticulum Stress by 2-Arylbenzofuran
Source: Int J Mol Sci. 2024 Jan 13;25(2):999. doi: 10.3390/ijms25020999 (PMC10816499; doi:10.3390/ijms25020999)
Supplement: Supplementary file 1 [file ijms-25-00999-s001.zip › ijms-2798366-supplementary.pdf]

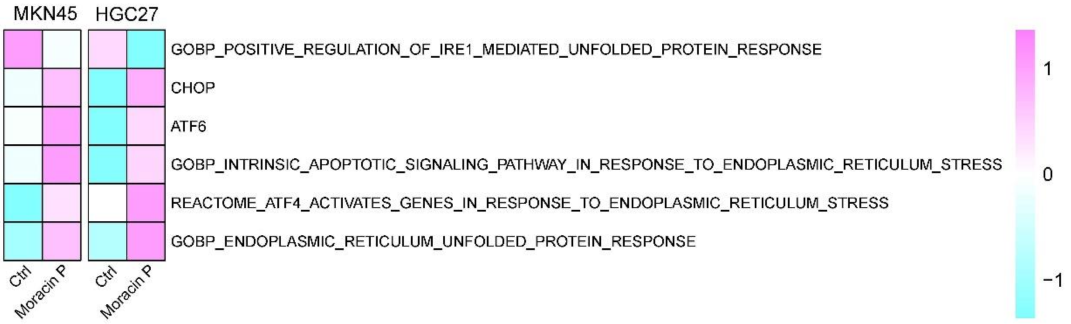

Figure S1. Gene Set Variation Analysis (GSVA) was conducted on pathways associated with the Unfolded Protein Response and Apoptosis, comparing cells treated with Moracin P to those treated with DMSO (used as the control, Ctrl).

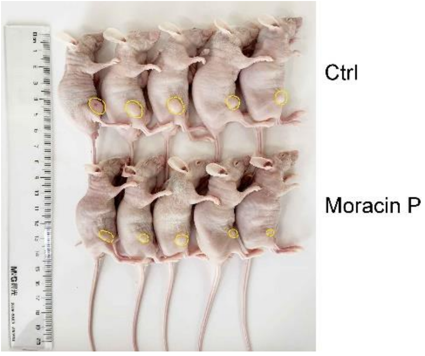

Figure S2. Images showcase tumors in the abdominal subcutaneous region of nude mice after intravenous (i.v.) injection of MKN 45 cells, followed by treatment with either Moracin P or DMSO (serving as the control, Ctrl).
